# Factors Associated With Digital Capacity for Health Promotion Among Primary Care Workers: Cross-Sectional Survey Study

**DOI:** 10.2196/63054

**Published:** 2024-12-20

**Authors:** Yining Wang, Hui Ren, Shaotan Xiao, Tian Meng, Shuyue Sun, Siyu Yu, Qing Liu, Fan Wang

**Affiliations:** 1 School of Public Health Fudan University Shanghai China; 2 Shanghai Pudong New Area Center for Disease Control and Prevention Shanghai China; 3 School of Accounting Shanghai University of International Business and Economics Shanghai China; 4 Fudan Development Institute Fudan University Shanghai China

**Keywords:** health promotion, digital capacity, primary care workers, Digital Capabilities Framework, online survey

## Abstract

**Background:**

Health education and promotion are recognized as effective strategies for fostering healthy ageing, reducing the disease burden, and addressing health inequalities, particularly when delivered through digital media. Primary care workers are often regarded as the key providers of these interventions. Despite the strong practical significance and substantial individual demand, the use of digital media for delivering health promotion practices was not widespread in China. One of the main challenges identified is the providers’ inadequate capacities. However, little is known about the digital capacity for health promotion among primary care workers.

**Objective:**

This study aimed to investigate the levels of digital capacity for health promotion and its associated factors among community health workers.

**Methods:**

A total of 1346 community health workers were recruited from across 47 communities in Shanghai, China, through cluster-stratified random sampling. The digital capacity for health promotion was measured using the revised version of the Digital Capabilities Framework. Web-based questionnaires were distributed to collect data from March 20 to March 29, 2024. Data were analyzed using descriptive statistics, independent t tests, one-way ANOVA, and linear hierarchical regression using Stata MP (version 17.0; StataCorp).

**Results:**

We included 1199 participants. Among them, 47.5% (570/1199) had high digital media use for more than 19.6 hours per week, whereas 31.8% (381/1199) demonstrated high digital media trust. The average level of digital capacity for health promotion was 16.71 (SD 2.94) out of 25 points. Demographics, digital media usage–related characteristics, perceived usefulness and usability, attitudes, and behaviors were significant predictors of the capacities, explaining 44.4% of the total variance. Master’s degree or above (β=.077; *P*=.013), perceived usability (β=.235; *P*<.001), attitudes toward digital media health promotion (β=.095; *P*=.002), and past digital media health promotion practices (β=.377; *P*<.001) had significantly positive associations with digital capacities for health promotion. However, senior (β=–.076; *P*=.008) or median (β=–.074; *P*=.01) titles had a significant negative association with capacity levels.

**Conclusions:**

A digitally capable workforce is required for primary health care systems to take full advantage of digital media health promotion. Therefore, solutions are necessary to achieve enhanced capacities among health professionals, including public health policy making, community empowerment, and individual practices.

## Introduction

As various countries worldwide are committed to fostering healthy ageing, reducing the disease burden, and narrowing health inequalities, health education and promotion have been identified as effective ways to achieve these health targets [1,2]. In both developed and developing countries, health promotion equips the public with the knowledge about the determinants of health and skills to cope with risk factors [3]. It plays a vital role in controlling the outbreaks of infectious diseases, curbing the rising number of chronic diseases, fostering healthy lifestyles, and ultimately contributing to improved health outcomes [4]. Furthermore, with the development of health-related digitization, social media platforms such as Instagram and Facebook have become some of the most important sources of health information acquisition, particularly among the younger population [5,6]. This situation lays a solid foundation for carrying out multiple health promotion activities through digital media, as it offers the opportunity to reach specific target populations, lowers the costs of implementation, and customizes interventions to the needs of different individuals [7].

Medical professionals play a paramount role in conducting health promotion activities [8]. Specifically, primary care workers are urged to engage in health promotion as part of their daily routines [2]. Globally, primary care professionals, such as general practitioners and nurses, serve as the gatekeepers of the residents’ health [9]. One of their imperative responsibilities entails the primary prevention of diseases before the first occurrence of undesirable symptoms, as opposed to secondary or tertiary prevention [7]. Even if a disease occurs, primary care workers can mobilize community resources to enhance community participation and reduce exposure to risk factors, thus enabling the efficient use of resources and fostering sustainability and resilience of the entire health system, simultaneously achieving cost savings and health gains [10]. Furthermore, primary care workers tend to have the most frequent contact with residents and obtain their deepest trust, which provides valuable insights that enable them to construct content that is relevant and accessible to the demands of the intended receivers [11,12].

China is one of the countries that have enacted to accelerate the implementation of health promotion through digital media. From the receivers’ perspective, individuals in China have demonstrated a growing need to acquire health knowledge through digital media [13]. This heightened health awareness creates an extensive need for preventive care and promotion services to address potential risk factors [14]. Studies have demonstrated that 67.13% of older adults have health promotion demands beyond the general level [15]. Furthermore, high internet prevalence in China has precipitated a surge of online health information–seeking behaviors [16]. The COVID-19 pandemic has further exacerbated this situation [6,13]. Current content and traditional face-to-face education have to some extent failed to meet the increasing need for health information via digital media [17]. From the providers’ perspective, relevant policies have been issued to support digitalized health promotion, particularly in primary health care (PHC) settings. In 2016, the State Council of the People’s Republic of China proposed the Healthy China 2030 Initiative, a critical guideline steering China’s health system development toward 2030, and incorporated health education into its strategies [18]. Since 2018, the government has provided funding to promote health promotion activities under the family physician contract, as family physicians are responsible for residents’ health maintenance and the effective delivery of public health education [5]. Subsequently, in 2022, supporting guidelines were proposed to increase the supply of high-quality health promotion activities through various media platforms [19].

However, despite the strong political wills and substantial individual demands, PHC professionals in China allocate insufficient time to health promotion practices, particularly those disseminated through digital channels [20,21]. Consequently, the prevalence of digitalized health promotion remains limited. A notable impediment to the adoption is providers’ lack of knowledge, awareness, and skills in using various digital media [22]. Several studies have investigated health care professionals’ promotion capacities, revealing that capacity levels may be affected by sex, age, educational level, occupation, training frequency, and familiarity with the policy [5,8]. Another strand of research concentrated on the measurement of digital capacities among health care workers. These studies identified potential influencing factors including sociocultural context [22], organizational support [23,24], individual motivation and attitudes [25,26], and the perceived usefulness and usability of digital technology [27,28]. Despite the abundant literature, there is a scarcity of similar studies on these 2 topics conducted in primary care settings in China. Moreover, the digital capacity for health promotion among health care workers, the integration of these 2 strands of literature, has not been systematically explored.

This study aims to investigate the digital capacity for health promotion in a representative sample of community health workers in Shanghai, China, and to identify the influencing factors. This study contributes to the literature on digital capacity and health promotion capacity among health care professionals. Furthermore, gaining knowledge about the status quo and correlates can suggest feasible strategies for continuously improving the quality of health promotion services, addressing health inequities, and building resilient health systems.

## Methods

### Research Design

This cross-sectional study aims to examine the current status of digital capacity for health promotion among community health workers based on the Digital Capabilities Framework (DCF) created by Health Education England and identify associated factors. To achieve this study objective, a survey was conducted. The survey included an anonymous web-based questionnaire to be completed by PHC providers in Shanghai, China.

### Recruitment

The study involved health care workers in community health centers located in the Pudong New Area, one of the largest and most demographically representative districts of Shanghai, China. As of 2023, Pudong New Area spanned an area of 1210 km^2^ and housed a population of 5.81 million residents, constituting approximately one-fourth of Shanghai’s total population [29]. Participants were recruited through cluster-stratified random sampling based on the predetermined inclusion criteria. All 47 communities within the Pudong New Area were chosen in the sampling process. Health care providers were stratified into 4 distinct layers according to their occupations (clinicians, public health physicians, nurses, and medical technicians). Following this categorization, the participants were randomly selected from the 4 layers, ensuring that each facility contributed more than 5 individuals from each occupational category. The inclusion criteria were as follows: (1) health workers providing health services in community health centers in the Pudong New Area of Shanghai, (2) able to understand the research purpose and voluntarily participate in the research, and (3) able to read and understand in Chinese.

**Figure 1:**
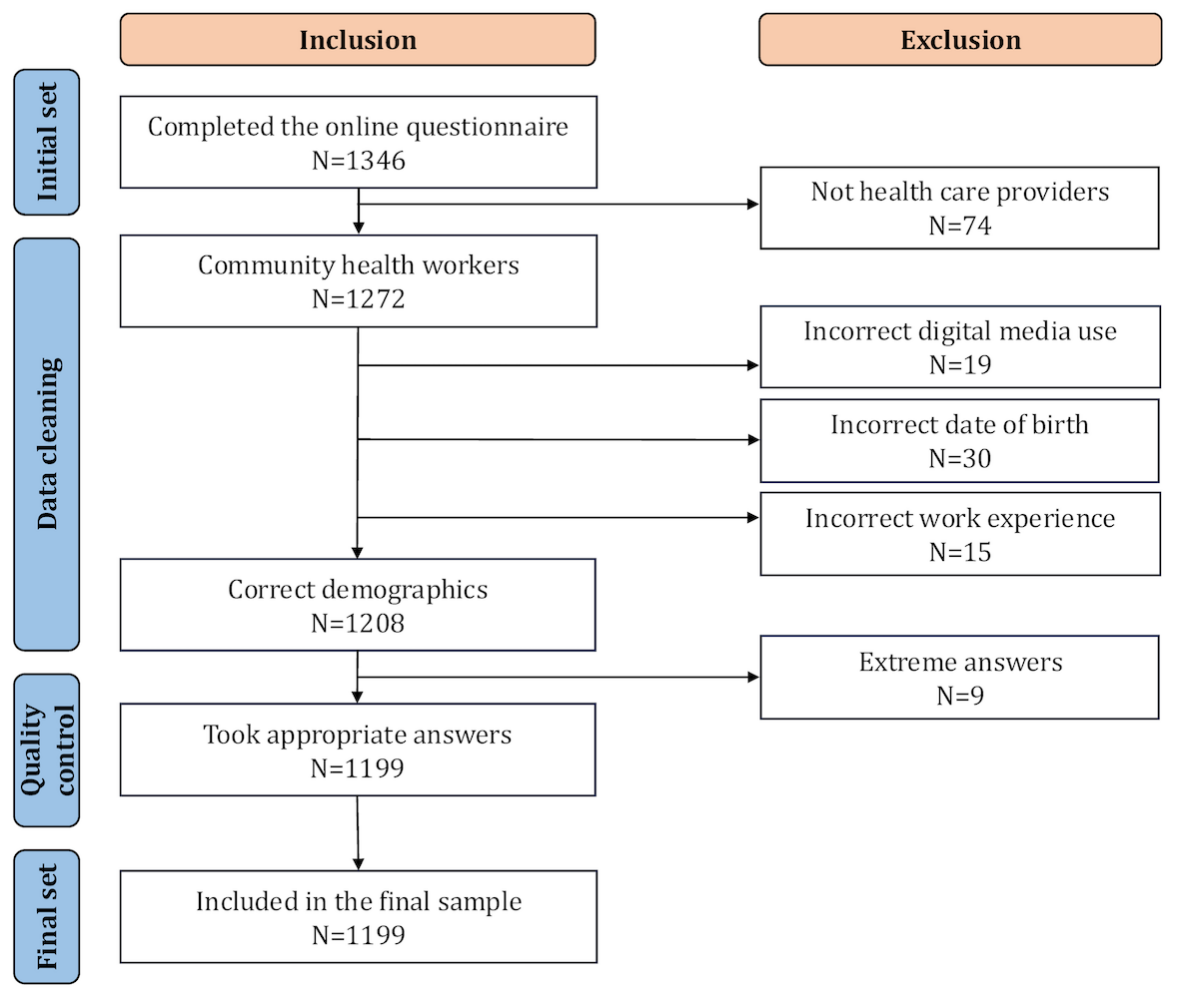
Flowchart for the sample inclusion.

A web-based questionnaire was developed and disseminated through the working groups in WeChat. It was completed by community health workers voluntarily using the Questionnaire Star platform from March 20 to March 29, 2024. A total of 1346 community health workers completed the questionnaire. To ensure data quality, we excluded incomplete or noncompliant questionnaires for quality control and concluded with 1199 participants for the final analysis, resulting in a response rate of 89.08% (1199/1346) (Figure 1).

### Ethical Considerations

This study received approval from the ethics review committee of the Shanghai Pudong New Area Center for Disease Control and Prevention (PDCDCLL-20240304-001). Participation was voluntary. Participants were informed of the purpose of the study and the possibility to opt out at any time. As a web-based survey, the completion and return of the self-administered questionnaire were regarded as providing informed consent. Measures were taken to ensure privacy and confidentiality by anonymizing the data, thus excluding any personal identifiers from the analysis. No compensation was offered.

### Measures

A web-based questionnaire was designed to measure the capacity levels and potential associates. Pilot surveys were conducted among 145 community health workers and health promotion practitioners to confirm the effectiveness, clarity, and readability of the questionnaire. The results were used for revision and optimization of the questionnaire design but were not included in the final analysis. After slight modifications, the survey comprised 6 sections.

#### Sociodemographics

The following participant demographics were collected: sex, age, seniority, educational level, occupation, technical title, digital media usage, and digital media trust. We asked respondents to identify how many hours they spend on various digital media platforms per week as a measurement for usage. Regarding digital media trust, respondents rated how they trust digital media sources of information on a 5-point Likert scale ranging from 1 (totally distrust) to 5 (totally trust) [30,31]. Following previous studies [27,32], individuals who achieved a score of the mean value or greater were defined as having “high digital media usage or trust”. In contrast, those who scored below average were regarded as having “low digital media usage or trust”.

#### Digital Capacity for Health Promotion

The digital capacity for health promotion was measured according to the DCF created by Health Education England [33] and previous studies adopting this framework [34]. It incorporates the following 5 items: communication, collaboration, and participation; teaching, learning, and self-development; information, data, and content literacy; creation, innovation, and research; and technical proficiency. Each of the 5 items was self-rated by the participants using a 5-point Likert scale ranging from 1 (strongly disagree) to 5 (strongly agree). The total score ranged from 5 to 25, with higher scores indicating higher levels of participants’ competencies. For evaluating the validity and reliability, we used Cronbach α for internal consistency and Kaiser-Meyer-Olkin (KMO) value for construct validity for each section. Cronbach α value was 0.94 and the KMO value was 0.90 in the final analysis.

#### The Usefulness of Digital Media Health Promotion

Previous theories have defined perceived usefulness as whether technology has practical value in the completion of tasks or in the attainment of goals, which has been identified as an important aspect affecting digital capacity [27,35]. Thus, a 5-point Likert scale with 4 items (from 1: strongly disagree to 5: strongly agree) was designed for the measurement. The final Cronbach α value was 0.89 and the final KMO value was 0.79 for this domain.

#### The Usability of Digital Media Health Promotion

Perceived usability is defined as whether learning and adopting a new technology is convenient, simple, and devoid of great difficulties, and has also been demonstrated to significantly influence digital capacity [36-38]. Two items on a 5-point Likert scale (from 1: strongly disagree to 5: strongly agree) were used to measure participants’ perceived usability. Cronbach α value was 0.82.

#### Attitudes Toward Digital Media Health Promotion

The participants’ subjective positive or negative attitudes toward the use of digital media for health promotion were assessed using 4 items measured on a 5-point Likert scale (from 1: strongly disagree to 5: strongly agree) [28,39]. Cronbach α value was 0.93, and the final set had a KMO value of 0.85.

#### Digital Media Health Promotion Behaviors

Past behaviors of carrying out health promotion via digital media were also measured, including the participants’ learning process, completed practice, and encouragement to colleagues. These 3 items are measured on a 5-point Likert scale (from 1: strongly disagree to 5: strongly agree). Cronbach α value was 0.90, and the KMO value was 0.75 in the final analysis.

### Statistical Analysis

The collected data were analyzed using Microsoft Excel (Microsoft Inc) and Stata MP (version 17.0; StataCorp). Descriptive statistics such as means and SDs were used for continuous variables, and frequencies and proportions were used for categorical variables. We conducted independent *t* tests and one-way ANOVAs to compare the differences in digital capacities concerning general characteristics, as homoscedasticity was ensured by Bartlett equal variances tests. Where ANOVAs showed significant differences, we performed Bonferroni tests as post hoc procedures. Linear hierarchical regression analysis was performed to examine the factors influencing digital capacities. Each categorical variable was dummy-coded in the regression model. The potential multicollinearity of variables was assessed using variance inflation factors. All the statistical tests were 2-sided, with *P* value of <.05 considered statistically significant.

## Results

### Sample Characteristics

The sociodemographic characteristics of the participants are shown in Table 1. A total of 1199 participants were included, of whom 81.6% (978/1199) were female. The average age was 39.34 (SD 7.70) years, and the average working experience was 17.20 (SD 9.03) years. An overwhelming majority of participants (950/1199, 79.2%) were university graduates with a bachelor’s degree, and 58.4% (700/1199) held median technical jobs. Among the participants, 27.5% (330/1199) were clinicians, 28.4% (341/1199) nurses, 24.7% (296/1199) public health physicians, and 19.4% (232/1199) medical technicians. Regarding digital media use and trust, 47.5% (570/1199) had high digital media use for more than 19.6 hours per week, whereas 31.8% (381/1199) demonstrated a high level of digital media trust.

**Table 1:** Sociodemographic characteristics of community health workers (N=1199).

Categories			Values
**Sex, n (%)**			
	Male	221 (18.43)	
	Female	978 (81.57)	
**Age (years), n (%)**			
	≤35	414 (34.53)	
	36-45	492 (41.03)	
	≥46	293 (24.44)	
**Seniority (years), n (%)**			
	≤10	335 (27.94)	
	11-20	427 (35.61)	
	≥21	437 (36.45)	
**Educational level, n (%)**			
	Associate’s degree or below	158 (13.18)	
	Bachelor’s degree	950 (79.23)	
	Master’s degree or above	91 (7.59)	
**Occupation, n (%)**			
	Clinician	330 (27.52)	
	Public health physician	296 (24.69)	
	Nurse	341 (28.44)	
	Medical technician	232 (19.35)	
**Technical title, n (%)**			
	Senior	106 (8.84)	
	Median	700 (58.38)	
	Primary	393 (32.78)	
**Digital media use, n (%)**			
	High	570 (47.54)	
	Low	629 (52.46)	
**Digital media trust, n (%)**			
	High	381 (31.78)	
	Low	818 (68.22)	

### Descriptive Statistics of the Study Variables

The results of descriptive statistics of the study variables are shown in Table 2. The participants’ average score of digital capacity for health promotion was 16.71 (SD 2.94) out of 25 points. Information, data, and content literacies had the highest value of 3.39 (SD 0.63), followed by teaching, learning, and self-development with a slightly lower value of 3.38 (SD 0.63). Creation, innovation, and research had the lowest score of 3.30 (SD 0.69). The mean score for the usefulness of digital media use was 3.49 (SD 0.61). Participants exhibited the highest agreement with the statement that digital media health promotion fostered the development of personal capacity (mean 3.59, SD 0.68), while lowest to its role related to job promotion. Regarding the usability of digital media health promotion, the score for this domain was 3.21 (SD 0.65) on average. Participants thought that it was easier to adapt to the potential changes than to acquire the related knowledge and skills. In addition, the domains of attitudes and behaviors had average scores of 3.57 (SD 0.59) and 3.29 (SD 0.64), respectively.

**Table 2:** Study variables among participants (N=1199).

Item		Mean (SD)	Range
**Digital capacity for health promotion**			
	Communication, collaboration, and participation	3.32 (0.66)	1-5
	Teaching, learning, and self-development	3.38 (0.63)	1-5
	Information, data, and content literacy	3.39 (0.63)	1-5
	Creation, innovation, and research	3.30 (0.69)	1-5
	Technical proficiency	3.32 (0.65)	1-5
	Total scores	16.71 (2.94)	5-25
**Usefulness of digital media health promotion**			
	Enhancement of professional value	3.57 (0.67)	1-5
	Development of personal capacity	3.59 (0.68)	1-5
	Promotion of technical title	3.35 (0.78)	1-5
	Assistance in carrying out the work	3.47 (0.68)	1-5
	Average score	3.49 (0.61)	1-5
**Usability of digital media health promotion**			
	Easy to acquire related knowledge and skills	3.16 (0.72)	1-5
	Easy to adapt to the changes in the work and life	3.26 (0.69)	1-5
	Average score	3.21 (0.65)	1-5
**Attitude toward digital media health promotion**			
	Meaningful to work on health promotion through digital media	3.63 (0.62)	1-5
	A great way to work on health promotion through digital media	3.60 (0.64)	1-5
	Not opposed to work on health promotion through digital media	3.62 (0.64)	1-5
	Relevant to my work	3.44 (0.67)	1-5
	Average score	3.57 (0.59)	1-5
**Digital media health promotion behavior**			
	Learnt about it through various channels	3.33 (0.67)	1-5
	Carried out in the scope of own work	3.25 (0.72)	1-5
	Encouraged colleagues to carry on	3.30 (0.70)	1-5
	Average score	3.29 (0.64)	1-5

### Differences in Digital Capacity for Health Promotion According to General Characteristics

Differences in digital capacity for health promotion according to the general characteristics of the participants are reported in Table 3. The results revealed significant differences according to age (*P*<.001), seniority (*P*<.001), occupation (*P*=.002), technical title (*P*<.001), and digital media trust (*P*<.001).

**Table 3:** Digital capacity for health promotion according to general characteristics of the participants (N=1199).

Categories		Mean (SD)		*t*/*F* test (*df*)	*P* value
**Sex**				0.19 (1, 1197)	.66
	Male	16.79 (2.99)			
	Female	16.69 (2.92)			
**Age (years)**				22.41 (2, 1196)	<.001
	≤35	17.42 (2.86)			
	36-45	16.53 (2.88)			
	≥46	16.00 (2.93)			
**Seniority (years)**				18.51 (2, 1196)	<.001
	≤10	17.41 (2.96)			
	11-20	16.75 (2.82)			
	≥21	16.13 (2.91)			
**Educational level**				1.42 (2, 1196)	.24
	Associate’s degree or below	16.54 (2.83)			
	Bachelor’s degree	16.69 (2.92)			
	Master’s degree or above	17.18 (3.27)			
**Occupation**				5.04 (3, 1195)	.002
	Clinician	16.20 (3.07)			
	Public health physician	16.76 (3.07)			
	Nurse	16.94 (2.79)			
	Medical technician	17.02 (2.69)			
**Technical title**				13.88 (2, 1196)	<.001
	Senior	16.18 (3.33)			
	Median	16.44 (2.81)			
	Primary	17.33 (2.94)			
**Digital media use**				0.31 (1, 1197)	.58
	High	16.66 (3.00)			
	Low	16.75 (2.88)			
**Digital media trust**				56.55 (1, 1197)	<.001
	High	17.62 (2.81)			
	Low	16.28 (2.90)			

Post hoc analyses using Bonferroni tests demonstrated that participant community health care workers aged 35 years or younger scored significantly higher than those aged between 36 and 45 years, which scored higher than those aged 46 years or older (all *P*<.05; Multimedia Appendix 1). Regarding seniority, participants who had worked for less than 10 years scored better (all *P*<.05). Nurses (*P*=.006) and medical technicians (*P*=.006) had higher digital capacities than clinicians. Furthermore, participants with a primary technical title scored better in their abilities than those with a median (*P*<.001) or senior title (*P*=.001).

### Influencing Factors of Digital Capacity for Health Promotion

Table 4 shows the results of the linear hierarchical regression. Demographics and digital media usage-related characteristics alone explained approximately 9.2% of the variance in digital capacity for health promotion, reaching statistical significance (*F*_14,1184_=8.619; *P*<.001). When including perceived usefulness and usability variables in model 3, the proportion of explained variance increased by 24.3% to an overall *R*^2^ value of approximately 33.6% (*F*_16,1182_=37.299; *P*<.001). Further including attitudes toward digital media health promotion into our model led to an additional increase in *R*^2^ of 3.8%. Finally, the inclusion of health promotion behaviors led to an increase in *R*^2^ by 7.1%, with an overall *R*^2^ of 44.4% (*F*_18,1180_=52.347; *P*<.001). Thus, our model significantly improved at each stage of the hierarchical process.

**Table 4:** Model parameters of the linear hierarchical regression model for digital capacity for health promotion (N=1199)^a^.

Model	Blocks	*R* ^2^	*F* test (*df*)	*P* value	△*R*^2^	△*F* test (*df*)	△*P* value
1	Demographics	0.054	5.594 (12, 1186)	<.001	0.054	5.594 (12, 1186)	<.001
2	Demographics + Digital media usage–related characteristics	0.092	8.619 (14, 1184)	<.001	0.039	25.387 (2, 1184)	<.001
3	Demographics + Digital media usage–related characteristics + Perceived usefulness and usability	0.336	37.299 (16, 1182)	<.001	0.243	216.133 (2, 1182)	<.001
4	Demographics + Digital media usage–related characteristics + Perceived usefulness and usability + Attitudes	0.373	41.334 (17, 1181)	<.001	0.038	70.702 (1, 1181)	<.001
5	Demographics + Digital media usage–related characteristics + Perceived usefulness and usability + Attitudes + Behaviors	0.444	52.337 (18, 1180)	<.001	0.071	150.580 (1, 1180)	<.001

^a^Variables included in the different hierarchical regression models are as follows: model 1 (sex, age group dummy coded, seniority group dummy coded, educational-level dummy coded, occupation dummy coded, and technical title dummy coded); model 2 (model 1 variables, digital media use group dummy coded, and digital media trust group dummy coded); model 3 (model 2 variables, perceived usefulness of digital media health promotion, and perceived usability of digital media health promotion); model 4 (model 3 variables, attitudes toward digital media health promotion); and model 5 (model 4 variables, digital media health promotion behaviors).

Table 5 reports the regression coefficients of the final hierarchical regression model. Of the control variables in blocks of demographics, those aged between 36 and 45 years had comparatively lower competencies than those aged 35 years or younger (*b*=–0.438, SE *B*=0.219, β=–.073; *P*=.046). Having a master’s degree or above (*b*=0.850, SE *B*=0.342, β=.077; *P*=.01) increased the capacity score to 0.085 in comparison with associate’s degree or below, whereas having a senior or median title decreased the score for –0.45 (*b*=–0.451, SE *B*=0.170, β=–.076; *P*=.008) and –0.76 (*b*=–0.761, SE *B*=0.301, β=–.074; *P*=.01) in comparison with a primary title, respectively. Furthermore, perceived usability (*b*=1.064, SE *B*=0.131, β=.235; *P*<.001), attitudes toward digital media health promotion (*b*=0.476, SE *B*=0.156, β=.095; *P*=.002), and digital media health promotion behaviors (*b*=1.727, SE *B*=0.141, β=.377; *P*<.001) were positively and significantly associated with higher scores in digital capacity for health promotion among community health workers.

**Table 5:** Regression coefficients for the final linear hierarchical regression model predicting the digital capacity for health promotion (N=1199)^a^.

			*b*		SE	β		*P* value		95% CI
**Sex (vs male)**										
	Female	-0.297		0.174		–.039	.088		–0.638 to 0.045	
**Age (years; vs ≤35 years)**										
	36-45	–0.438		0.219		–.073	.046		–0.867 to –0.009	
	≥46	–0.589		0.315		–.086	.061		–1.207 to 0.028	
**Seniority (years; vs ≤10 years)**										
	11-20	0.405		0.227		.066	.075		–0.041 to 0.851	
	≥21	0.471		0.306		.077	.12		–0.128 to 1.071	
**Educational level (vs associate’s degree or below)**										
	Bachelor’s degree	0.223		0.215		.031	.30		–0.198 to 0.645	
	Master’s degree or above	0.850		0.342		.077	.013		0.178 to 1.521	
**Occupation (vs clinician)**										
	Public health physician	–0.201		0.195		–.030	.30		–0.584 to 0.182	
	Nurse	0.137		0.201		.021	.50		–0.258 to 0.532	
	Medical technician	0.141		0.206		.019	.49		–0.263 to 0.544	
**Technical title (vs primary)**										
	Median	–0.451		0.170		–.076	.008		–0.784 to –0.118	
	Senior	–0.761		0.301		–.074	.012		–1.352 to –0.171	
**Digital media use (vs low)**										
	High	–0.174		0.131		–.030	.19		–0.431 to 0.083	
**Digital media trust (vs low)**										
	High	0.205		0.146		.033	.16		–0.081 to 0.491	
**Perceived usefulness**		0.173		0.143		.036	.23		–0.107 to 0.452	
**Perceived usability**		1.064		0.131		.235	.23		–0.107 to 0.452	
**Attitudes toward digital media health promotion**		0.476		0.156		.095	.002		0.169 to 0.783	
**Digital media health promotion behavior**		1.727		0.141		.377	<.001		1.451 to 2.003	
**Constant**		5.644		0.549		N/A^b^	<.001		4.567 to 6.721	

^a^*R*^2^=0.444, adjusted *R*^2^=0.436, *F*=52.35, and *P*<.001.

^b^N/A: not applicable.

## Discussion

### Principal Findings in Relation to Previous Studies

This study examined the current levels of digital capacity for health promotion among community health workers in China and identified its influencing factors. Our findings suggested that the participants’ average level of digital capacity for health promotion was 16.71 (SD 2.94) out of 25 points. Demographics, digital media usage–related characteristics, perceived usefulness and usability, attitudes, and behaviors were significant predictors of the capacity. Those with higher educational levels, perceived usability, more positive attitudes, and more frequent behaviors had significantly higher digital capacities for health promotion. However, the technical titles were negatively associated with capacity scores. In this digital era, strengthening the digital competencies of health care providers contributes to increasing access to health information, thereby improving the quality of practice, and raising public awareness of healthy lifestyles [26]. Furthermore, it has also demonstrated potential positive impacts on the health equality and resilience of the entire health system when facing public health emergencies and increasing chronic disease burdens.

Based on the success of the prior literature, our study specifically focused on the capacity levels for health promotion among health care providers. Previous studies have established different terms to refer to the concept of digital capacity, such as eHealth literacy and digital health competence, and have formulated operationalized frameworks for measurement and comparison [40,41]. However, few studies assessed digital capacity in the health promotion process, an important issue gaining increasingly wider popularity in the digital era [42-44]. Moreover, studies have been conducted in PHC settings to investigate providers’ skill development in health education and health promotion activities but were predominantly focused on specific programs, notably diabetes management, mental health care, and cancer prevention [17,45,46]. Evidence regarding the comprehensive assessment of PHC workers’ capacity levels is scarce. In this context, we modified the DCF to incorporate the health promotion process into the measurement scale and observed that the average score among community health workers was 16.71 (SD 2.94) out of 25 points. Information, data, and content literacy was the highest item scoring 3.39 (SD 0.63), whereas creation, innovation, and research was the lowest item scoring 3.30 (SD 0.69). When compared with similar studies measuring health care providers’ digital capacity or health promotion capacity, our study reported a relatively lower score when focusing specifically on the integration of these 2 capacities [25,27,47]. This was evidenced by the achieved scoring of only 66.9% (16.71/25), significantly lower than similar studies in China that achieved close to or above 80% of total scores in different scales [5,48]. Furthermore, studies in other countries also confirmed the relatively high level of wished scoring, with most studies conducted in resource-limited regions with limited internet penetration, unlike Shanghai [37,49,50]. This comparison indicates significant room for improvement in the digital capacity of community health workers when conducting health promotion activities in China.

Regarding the determinants, our results indicated that 5 blocks, namely, demographics, digital media usage–related characteristics, perceived usefulness and usability, attitudes, and behaviors, were all significant predictors. These findings are consistent with those of previous studies investigating factors associated with digital health literacy conducted elsewhere around the globe [50-52]. In our final regression model, attainment of a master’s degree or above (β=.077; *P*=.01) was correlated with significantly greater capacity levels, possibly owning to their reception of past formal training curriculums and proficiency in simplifying intricate health concepts [53]. Conversely, having a senior (β=–.076; *P*=.008) or median title (β=–.074; *P*=.01) was significantly negatively associated with the score. This could possibly be elucidated by recent considerations of health education practices in job promotions among community health workers in China, motivating workers with primary titles to enhance their skills [54]. Furthermore, the propensity of the younger generation to engage with digital media in a proactive way might also contribute to this trend [55]. In addition to sociodemographics, perceived usability (β=.235; *P*<.001) was also a significant correlate, since health care providers tend to be more motivated and enthusiastic when having mastered the skills and knowledge of digital media health promotion [27]. They are more confident in performing promotional practices using digital tools, thus attaining a more desirable capacity level. Furthermore, digital capacity was influenced by current attitudes (β=.095; *P*=.002) and existing behaviors (β=.377; *P*<.001). This could be attributed to the fact that positive attitudes toward digital media fostered health workers’ involvement in technology and their search for digital information [56]. By adopting digital media to source reliable data, synthesize understandable content, and disseminate it to various patients during the process of health popularization, their competencies can be progressively cultivated and strengthened.

### Implications

These findings have valuable implications for public health policy making, community empowerment, and individual practices. These significant predictors can serve as important indicators for delivering targeted interventions to strengthen the PHC providers’ digital capacity for health promotion in China. Specifically, a digitally capable workforce in PHC settings ensures equitable, accessible, and high-quality health promotion programs delivered through various digital media channels [10]. This improves the coverage of the most vulnerable or disadvantaged population groups, thereby contributing to a reduction in health inequalities [57]. Therefore, solutions aligned across policy-level interventions, community-level inputs, and individual-level efforts are urgently required. The results demonstrated that positive attitudes and previous engagement in digital health promotion were crucial facilitators fostered by the strategies at all 3 levels. Lower educational levels and perceived usability could be potential barriers, requiring measures at the community level.

At the policy level, relevant policies can be enacted to encourage health workers to use digital tools when carrying out health promotion activities. Strategies targeting economic incentives or other rewards can foster health workers’ attitudes toward digital media health promotion, thus obtaining more desirable capacity levels [57]. Moreover, it is worth noting that recent years have witnessed the expansion of nurses’ roles worldwide, particularly in health promotion responsibilities aimed at preventing chronic diseases and addressing unhealthy lifestyles [58]. The central government should thus commit to increasing the supply of nurses and enhancing their competencies by issuing relevant policies and guidelines. At the community level, resources should be invested to provide the necessary training programs to the workforce. Community health centers should develop purposeful training plans to familiarize health care workers with the use of digital tools and related theories, thereby obtaining higher usability levels [59]. Furthermore, a supportive organizational environment is crucial for translating policies into management and operational mechanisms. Possible approaches include advocating for benefits, addressing challenges encountered, and fostering implementation. By encouraging PHC workers to participate in digital health promotion activities, the institutional service capacity can also be strengthened and empowered [60]. At the individual level, community health workers should maintain positive attitudes and proactively engage in digital media health promotion activities to gain elevated capacities through multiple practices. They would in turn benefit from these policies and community-based measures by gaining a better understanding of the significance of health education activities and take advantage of various training programs to fill their capacity gaps.

### Limitations and Directions for Future Research

Our study has several limitations. First, all of the study variables were self-reported by the participants through web-based questionnaires, with the possibility of reporting bias occurring during the survey. Future studies can benefit from objectively evaluating the digital capacity of community health workers. Second, in this study, we primarily concentrated on the effects of individual factors fully considering broader contextual factors at the community and social levels. As previous studies have confirmed the connection between health workers’ digital capacities and various cultural, economic, and political characteristics, more efforts should be invested in assessing factors at different levels simultaneously. Third, this study included only health workers in community health centers located in Shanghai, a tier-1 megacity in China. Thus, the results cannot be generalized to the entire population of health care providers nationwide. Future studies should be conducted with a group of participants representative of the nationwide health workforce nationwide to enhance the generalizability of the findings. Finally, this study examined the digital capacity of community health workers, without investigating further impacts on patients. Further research is required to evaluate the effects of providers’ elevated capacities regarding improving patients’ responsiveness and health outcomes.

### Conclusions

Health promotion through digital media, particularly in primary care settings, has significant implications for improving the quality of health promotion services, addressing the increasing disease burden, and fostering the resilience of the health system. However, its implementation is still not extensive in primary care facilities in China, partly owing to the lack of relevant capacity among health workers. Our study found that the average level of digital capacity for health promotion among community health workers was 16.71 (SD 2.94) out of 25 points. Demographics, digital media usage–related characteristics, perceived usefulness and usability, attitudes, and behaviors were significant predictors of the capacity, explaining 44.4% of the total variance. Future interventions are urgently required at the policy, community, and individual levels to collectively cultivate primary care professionals with elevated capacities.

## Appendix

Multimedia Appendix 1 Post hoc analyses using Bonferroni tests.

## Abbreviations

DCF: Digital Capabilities Framework
KMO: Kaiser-Meyer-Olkin
PHC: primary health care
