# Supplementary material for: Factors Associated With Digital Capacity for Health Promotion Among Primary Care Workers: Cross-Sectional Survey Study
Source: J Med Internet Res. 2024 Dec 20;26:e63054. doi: 10.2196/63054 (PMC11699497; doi:10.2196/63054)
Supplement: Multimedia Appendix 1 [file jmir_v26i1e63054_app1.docx]

**Multimedia Appendix 1**

**Table S1. Bonferroni tests of different age groups.**

|  | ≤35 | 36-45 |
| --- | --- | --- |
| 36-45 | -0.89 |  |
|  | (<0.001) |  |
| ≥46 | -1.42 | -0.53 |
|  | (<0.001) | (0.041) |

*P* values in parentheses

**Table S2. Bonferroni tests of different occupations.**

|  | Clinician | Public Health Physician | Nurse |
| --- | --- | --- | --- |
| Public Health Physician | 0.57 |  |  |
|  | (0.093) |  |  |
| Nurse | 0.75 | 0.18 |  |
|  | (0.006) | (1.000) |  |
| Medical Technician | 0.82 | 0.26 | 0.08 |
|  | (0.006) | (1.000) | (1.000) |

*P* values in parentheses

**Table S3. Bonferroni tests of different seniority groups.**

|  | ≤10 | 11-20 |
| --- | --- | --- |
| 11-20 | -0.65 |  |
|  | (0.006) |  |
| ≥21 | -1.28 | -0.62 |
|  | (<0.001) | (0.010) |

*P* values in parentheses

**Table S4. Bonferroni tests of different technical titles.**

|  | Senior | Median |
| --- | --- | --- |
| Median | 0.26 |  |
|  | (1.000) |  |
| Primary | 1.15 | 0.89 |
|  | (0.001) | (<0.001) |

*P* values in parentheses
